# Classification needed of the private health sector

**DOI:** 10.2471/BLT.23.290869

**Published:** 2023-11-01

**Authors:** Muhammad Naveed Noor, Aya Thabet, Hassan Salah, David Clarke, Zafar Mirza

**Affiliations:** a Institute for Global Public Health, Department of Community Health Sciences, University of Manitoba Winnipeg, R065-771 McDermot Avenue, Winnipeg, Manitoba R3E OT6 Canada.; b World Health Organization Regional Office for the Eastern Mediterranean, Cairo, Egypt.; c Primary Health Care Special Programme, World Health Organization, Geneva, Switzerland.; d School of Universal Health Coverage, Shifa Tameer-e-Millat University, Islamabad, Pakistan.

The World Health Organization (WHO) acknowledges that the absence of a common definition of the private health sector may hinder a comprehensive understanding of its ubiquity.^1^ Physicians, nurses, pharmacists, midwives and various other health professionals outside the public health facilities are parts of a larger entity known as the private health sector, yet no formal taxonomy exists to identify domains, their functionality and the hierarchy between them.

The global health community confronts several health challenges such as an increase in the noncommunicable disease burden and antimicrobial resistance.^2^ Furthermore, epidemics and pandemics are projected to increase,^3^ and these outbreaks not only cause morbidity and mortality but also affect our social and economic life.^4^^–^^6^ These growing health challenges require that our health systems be well-equipped to deliver optimal health-care services, and engagement with the private health sector can be highly effective to deliver equitable health care.

The private health sector runs parallel to public health-care facilities, and plays an important role in the provision of health care. In many low-and middle-income countries, due to low public health spending, governments and patients have been increasingly reliant on the private health sector.^7^ Consequently, WHO and Member States are placing more emphasis on private sector engagement to achieve universal health coverage. Indeed, due to population increase, insufficient resources of the public sector, and/or less health spending in many low- and middle-income countries, advocating for effective private sector engagement seems reasonable.^8^

The private health sector is vast and evolving; its diversity makes it complex, since it may be profit-making or not-for-profit, formal or informal. Therefore, mapping out elements of the private health sector and the unique roles they play in the delivery of health care is critical for effective private sector engagement. Doing so has two benefits for research: a better theoretical understanding of the private health sector, and an increased accuracy and generality of empirical research. In health-care services literature, primary health care, immunization, family planning, oral health services, human immunodeficiency virus (HIV) prevention and treatment and digital health have all been noted as services that the private health sector enables. Because the effectiveness of any single element (that is, for-profit primary health-care clinics) of the private health sector may not be generalizable to other elements (not-for-profit primary health-care clinics), research performance data on this element may not portray an accurate picture of the performance of this sector.

Despite the need to conceptualize and classify the private health sector, hardly any attempts have been made to do so, and the ontological issues of the private health sector remain. Therefore, achieving consistency in empirical research and clarity in private sector engagement might be difficult. Just as biodiversity has been very well explained through taxonomy (the science and practice of the classification of organisms), public health researchers need to think along those lines. Conceptual clarity of the diversity of the private health sector will help researchers to analyse the utility of its various components, which can further inform policy-making and practice.

Health systems are like mechanical systems consisting of several parts working together in a synchronized manner, monitored by sensors and controllers. In a similar vein, the private health sector is constituted by individuals, enterprises and organizations that are neither owned nor directly controlled by governments, and are involved in the provision of health services. As the sector evolved, it continued to embrace new elements outside public health enterprises. Health-care provision, research and development, manufacturing and retail are different but interlinked streams that work together to make up the private health sector, which means that lumping these streams together as one single entity is conceptually inappropriate.

A starting point could be to collect data about the full range of professionals, enterprises and organizations in health care. Through the data, the private health sector could be conceptually broken down into macro, meso and microelements; and within each domain, the composition, functionality and relationality of various elements of the private health sector must be analysed. Similar to the organization of biodiversity through phylogenetic trees, a graphical representation of the classification of the private health sector could be performed, which can also lead to the identification of major domains of health-care professions and hierarchies within those domains. This foundational work can prove highly effective in determining the over- or under-utilization of different components of the private health sector.
